# Evaluation of Important Molecular Pathways and Candidate Diagnostic Biomarkers of Noninvasive to Invasive Stages in Gastric Cancer by In Silico Analysis

**DOI:** 10.1155/2021/5571413

**Published:** 2021-05-07

**Authors:** Sara Tutunchi, Saeedeh Akhavan, Ahmad Bereimipour, Sayyed Mohammad Hossein Ghaderian

**Affiliations:** ^1^Department of Medical Genetics, Shahid Sadoughi University of Medical Sciences, Yazd, Iran; ^2^Department of Biology, School of Basic Sciences, Science and Research Branch, Islamic Azad University, Tehran, Iran; ^3^Department of Stem Cells and Developmental Biology, Cell Science Research Center, Royan Institute for Stem Cell Biology and Technology, ACECR, Tehran, Iran; ^4^Faculty of Sciences and Advanced Technologies in Biology, University of Science and Culture, Tehran, Iran; ^5^Urogenital Stem Cell Research, Shahid Beheshti University of Medical Sciences, Tehran, Iran

## Abstract

Gastric cancer affects millions of people each year; it is the fifth deadliest cancer globally. Due to failure to perform routine tests such as endoscopy, it is usually diagnosed in the invasive stages. Therefore, finding diagnostic biomarkers in blood can help to speed up the initial diagnosis of cancer. This study aimed to find appropriate diagnostic biomarkers in the extracellular matrix of noninvasive to invasive stages of gastric cancer patients, using bioinformatics analysis. First, we selected the appropriate datasets from the GEO database. We evaluated the genes' signaling pathways, biological processes, and molecular functions. More accurately, we assessed the genes, in which their protein products are released into the extracellular matrix; we evaluated their protein network. Then, we validated the candidate proteins in the GEPIA and TCGA databases. The extracellular matrix, tyrosine kinase receptors, and immune response pathways are effective factors, which are related to the highly expressed genes and metabolism; cell cycle pathways are also impressive on low-expression genes. 69 highly expressed proteins are released into the extracellular matrix. After drawing the protein network, 5 proteins were selected as more suitable candidates for further studies. These proteins' expression significantly increases in the human samples, and the survival chart showed up to about 80% mortality in the individuals over time. With integrated bioinformatics analysis, BGN, LOX, MMP-9, SERPINE1, and TGFB1 proteins have been selected as suitable diagnostic biomarkers for noninvasive to invasive stages of gastric cancer. Further studies are needed to evaluate more precise mechanisms between these proteins.

## 1. Introduction

Gastric cancer is one of the most important and life-threatening cancers in the world. It is the fifth common cancer, which affects about 1.5 million people in the world annually. On the contrary, it has several risk factors; one of the most important is *Helicobacter pylori* infection, which is a common disease among people worldwide [1, 2].

The patients' age is a treatment challenge. The average age of gastric cancer patients is over 40; even in many countries, it starts from the age of 70 [3, 4]. Also, developing countries do not make timely diagnosis for gastric cancer due to the lack of regular use of diagnostic tests to examine the gastrointestinal tract, such as endoscopy and photofluorography; so the cancer becomes apparent in the invasive stages [5].

Therefore, the initial diagnosis can be checked using more straightforward screening methods, by finding biomarkers in patients' blood. Then, diagnostic tests can be performed. In this regard, bioinformatics techniques are an effective way to study and select genes and their protein products in various diseases [6, 7], such as cancer; they have made acceptable progress. This study analyzed the gene expression profile in noninvasive to invasive stages of gastric cancer using appropriate datasets. Finally, we further investigated the genes and protein products, which play a significant role in the extracellular matrix.

## 2. Materials and Methods

### 2.1. Selecting the Appropriate Dataset

**Figure 1:**
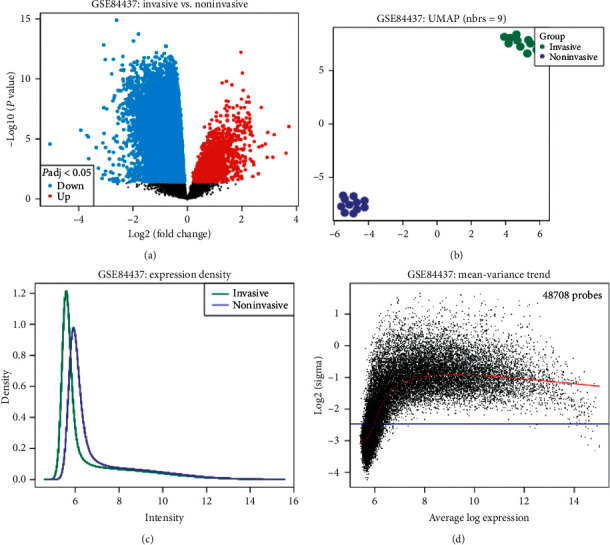
The GSE84437 dataset selected data of the noninvasive and invasive stage of gastric cancer. (a) The gene expression profile in the volcano diagram. Red is for high-expressed genes, and blue is for low-expressed genes. (b) PCA diagram is drawn to show the quality of the samples. (c, d) The amount of accumulation and mean expression of genes based on LogFC.

Using the GEO database, we selected the appropriate dataset. GSE84437 microarray dataset contains 433 samples, which have been classified according to the pathological staging, from the primary to the invasive stage of the disease. The platform of this dataset is GPL6947 Illumina HumanHT-12 V3.0 expression BeadChip. This section examined the patients with gastric cancer in the two separate groups of noninvasive and invasive stage. In Figure 1, one can see more information about this database.

### 2.2. Preparation of the Data for Bioinformatics Analysis

Using the GEO database and the GEO2R tool, we isolated the gene expression profiles between the noninvasive and invasive stage of gastric cancer. Then, we uploaded the genes into an Excel file and categorized the high- and low-expressed genes. In this part, *P* < 0.05 was considered as the statistically significant level; no limit was set for LogFC. No expression differentiation ranges were applied to the genes clustering in the study.

### 2.3. Finding Signaling Pathways and Gene Ontologies

For this step, we uploaded the high- and low-expressed genes separately into the Enrichr database: KEGG and Reactome libraries were used to isolate the signaling pathways from the gene clusters. To examine the gene ontology, we used the GO library in the Enrichr and PANTHER databases. Then, we used the ShinyGO database to draw the interaction network of the gene ontology.

### 2.4. Measuring the Relationship between the Protein Products

We used the STRING database to study the protein products of the genes. Then, we examined the proteins, which were most related to each other, at the center of the protein network.

### 2.5. Confirmation of Candidate Genes in the Gastric Cancer Samples

To more accurately confirm the selected genes from the previous steps and especially the relationship between the protein network, the final genes were evaluated in the GEPIA database; it uses the TCGA and GTEx databases to examine the gene expression in patients' samples. Survival and gene expression diagrams were drawn as box plot; they were illustrated by the GEPIA database.

## 3. Results

### 3.1. Examination of the Expression of Differential Genes in the Period between the Noninvasive and Invasive Stage of Gastric Cancer

The categorized genes' expression profile showed 1347 high-expressed and 3281 low-expressed genes. For this evaluation, 10 patients in the noninvasive and invasive stage were selected based on the pathological stage; their expression profiles were evaluated. The data quality and classification performed in the PCA diagram are shown in Figure 1. The highest gene expression was related to *MYH11* (LogFC: 2.925856), *THBS4* (LogFC: 2.6688873), *COL8A1* (LogFC: 2.5590963), *CNN1* (LogFC: 2.43792), and *GREM1* (LogFC: 2.4140408). The lowest gene expression was related to *PGC* (LogFC: −4.2971564), *LIPF* (LogFC: −3.534097), *PGA5* (LogFC: −3.4814338), *GKN1* (LogFC: −3.4252579), and *PGA3* (LogFC: −3.3898602).

Signaling pathways of the extracellular matrix, tyrosine kinase receptors, immune response, metabolism, and cell cycle were significantly observed in the invasive and noninvasive stage of gastric cancer.

**Figure 2:**
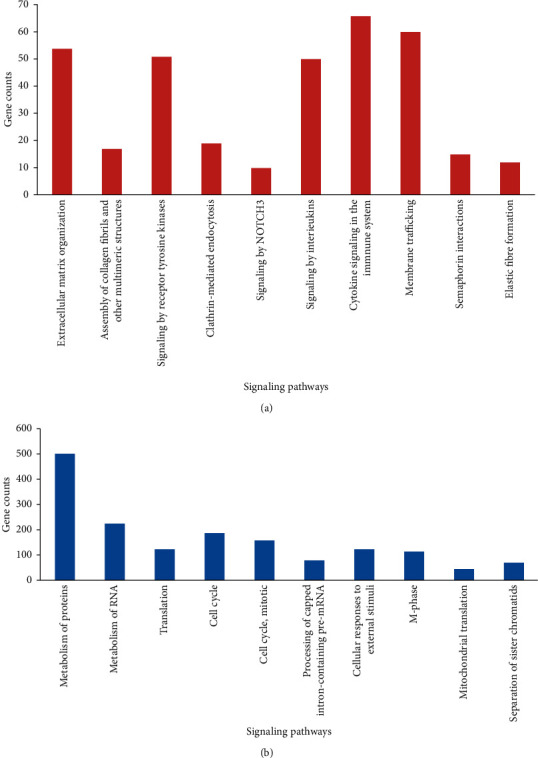
The number of involved genes in essential signaling pathways. (a) Upregulated genes. (b) Downregulated genes.

After examining the high- and low-expressed genes in the Enrichr database, the following factors were found to be more prominent: the extracellular matrix, assembly of collagen fibrils and other multimeric structures, receptor tyrosine kinases, clathrin-mediated endocytosis, signaling by NOTCH3, interleukins and immune system, membrane trafficking, and semaphorin interaction signaling pathways in the high-expressed genes and the cell cycle, metabolism of proteins, translation, processing of capped intron-containing pre-mRNA, cellular responses to external stimuli, mitochondrial translation, and separation of sister chromatids' signaling pathways in the low-expressed genes.  Figure 2 shows the number of genes involved in each of the signaling pathways separately.

### 3.2. Gene Ontology in Noninvasive and Invasive Gastric Cancer

**Figure 3:**
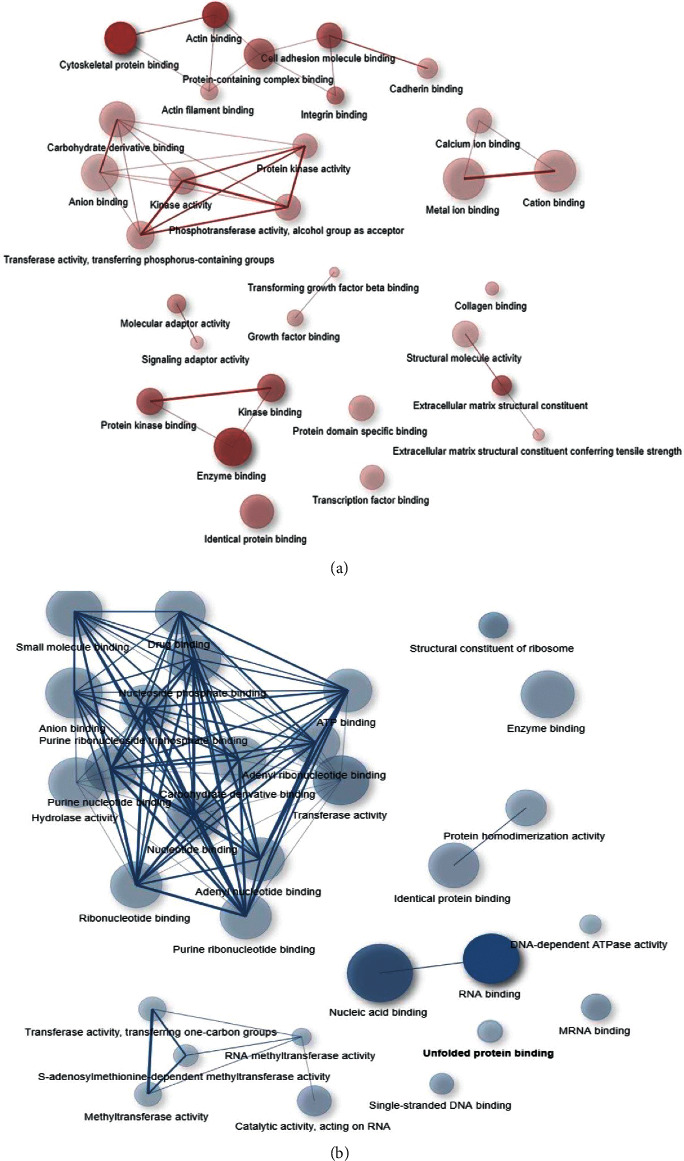
Molecular functions between the high- and low-expressed genes are shown in a network. The intensity of color and the circles' size indicate the significance of the molecular functions. (a) Upregulated genes. (b) Downregulated genes.

Different approaches have examined the genes' nature. In this section, genes were evaluated by three approaches including biological processes, molecular functions, and cellular components. As one can see in Table 1 and Figure 3, biological processes and molecular functions were studied with more focus. Anatomical structure morphogenesis, regulation of the cellular component organization, regulation of the developmental process, actin filament-based process, multicellular organismal process, and vasculature development pathways in biological processes and cytoskeletal protein binding, actin binding, enzyme binding, extracellular matrix structural constituent, cell adhesion molecule binding, and kinase binding in molecular functions with high expression were observed among them.

**Figure 4:**
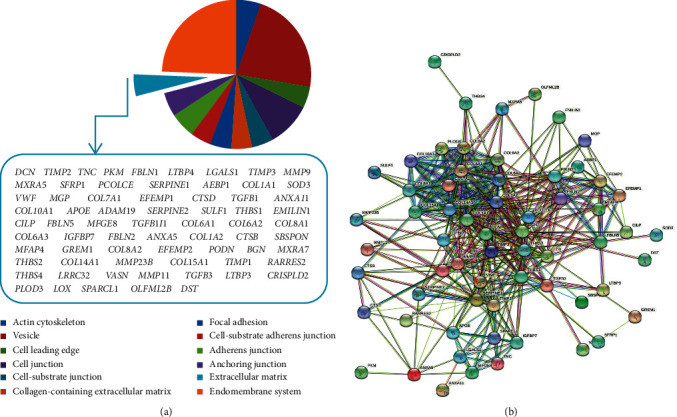
(a) The genes whose protein products were released into the extracellular matrix were isolated. (b) The interaction network between proteins in the extracellular matrix was identified.

RNA processing, organonitrogen compound biosynthetic process, mitotic cell cycle, cellular response to stress, and peptide metabolic process in biological processes and nucleic acid binding, structural constituent of the ribosome, transferase activity, mRNA binding, and small-molecule binding in molecular functions were observed in the bioinformatics data of low-expressed genes. In the cellular components, the focus was on the 69 involved genes in the extracellular matrix. They have been shown in Figure 4(a).

### 3.3. The Correlation of the Extracellular Matrix Protein Network in the Noninvasive to Invasive Gastric Cancer

In this part of the study, a significant protein network was obtained between the genes, in which their protein products were released into the extracellular matrix. This protein network had 69 nodes, 380 edges, and protein-protein interaction enrichment (*P* value: <1.0*E* − 16). Based on the analyses performed by the STRING database, it was found that these genes play an effective role in the ECM-receptor interaction, focal adhesion, TGF-beta, and the PI3K-Akt signaling pathway. In Figure 4(b), one can see more detailed information on how proteins are related and their associated pathways. Accordingly, in this section, the proteins with the most connection with other proteins in the network were selected; the selected proteins are as follows: BGN, LOX, MMP-9, SERPINE1, and TGFB1.

### 3.4. Candidate Genes Significantly Showed Higher Expression in the Patients with Gastric Cancer

**Figure 5:**
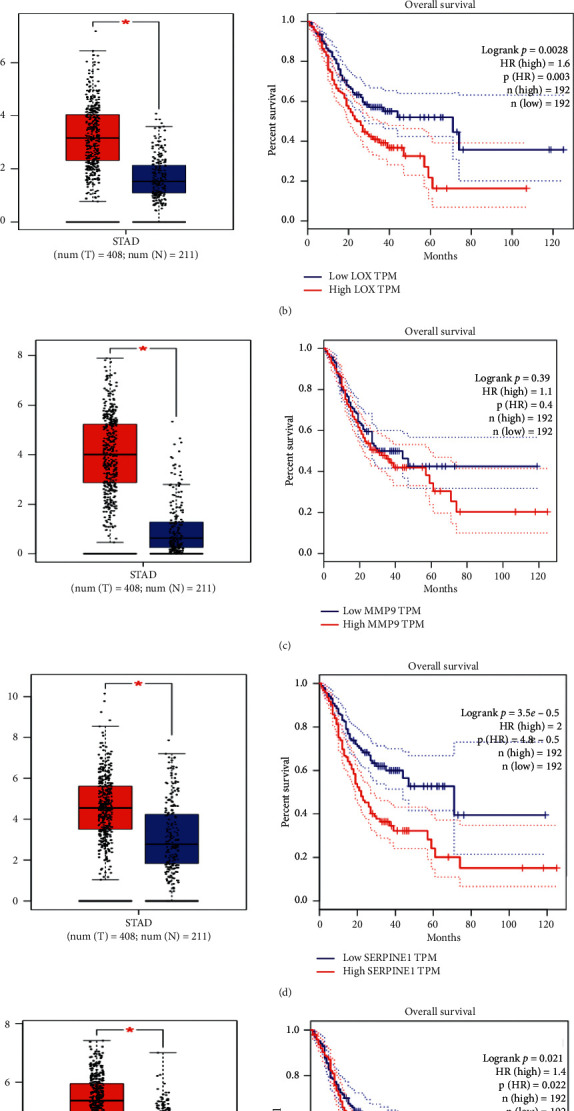
Expression of candidate genes in gastric cancer patients and healthy individuals is shown in a box plot. Survival charts have also been drawn for each of the genes and their protein products, suggesting that these genes significantly increase mortality over time. (a) BGN; (b) LOX; (c) MMP9; (d) SERPINE1; (e) TGFB1.

We evaluated the identification of hub genes and protein products, which play a significant role in gastric cancer's invasive state using the GEPIA database. The expression of BGN, LOX, MMP-9, SERPINE1, and TGFB1 proteins was significantly higher in the samples of gastric cancer patients compared to the healthy people. The survival chart for cited proteins showed 60–80% mortality increment in patients with gastric malignancy (Figure 5); it indicated the importance of these genes' role.

## 4. Discussion

Identifying the gastric cancer biomarkers can improve the process of diagnosis and treatment. Usually, a high percentage of people in a community do not undergo endoscopic and photofluorography tests, unless they have a specific digestive problem [8, 9]. Also, because the average age of patients with gastric cancer is 40, many people do not desire to perform these tests routinely [10]. For this reason, careful examination and biomarkers' selection in the body secretions such as blood, saliva, or urine can be helpful in the first step of analysis in the gastric cancer candidates.

In this study, in the first step, bioinformatics analysis could help to predict the candidacy of effective diagnostic biomarkers in gastric cancer. In addition, the gene expression profile of individuals was evaluated in the noninvasive to invasive stage of gastric cancer. In the first step, the associated signaling pathways of gastric cancer invasion were obtained.

One of the important signaling pathways of cancer cell metastasis is the NOTCH3 pathway, which is one of the main angiogenesis actors, but this gene works in many other ways and can facilitate the invasion of cancer cells [11]. The study by Kang et al. showed a direct link between NOTCH3 and Jagged2; NOTCH3 is effective in the development and recurrence of gastric cancer [12]. A study by Wu et al., which assessed patients with gastric cancer, showed that 4 NOTCH receptors play an effective role in stimulating gastric cancer tumor cells; they can be selected as a prognostic biomarker in gastric cancer [13]. Another study by Du et al., a meta-analysis of 1547 gastric cancer patients and 450 control samples, showed higher expression of NOTCH1, NOTCH3, and Jagged1 in the cancer samples than healthy individuals [14]. In general, more studies are needed to find NOTCH3-related pathways in increasing gastric cancer invasion.

One of the most attractive signaling pathways observed in this study was the semaphorin pathway. The semaphorin protein family plays a major role in axonal guidance in neurons. Still, several studies have shown that these neurotransmitters can also play a role in the growth, division, and migration of cancer cells. In a survey by Miyato et al., it was shown that increased SEMA3C expression is directly related to the increased invasion of gastric cancer cells [15]. Maejima et al.'s study also indicated that increased SEMA3E expression could play a key role in the development and metastasis of the gastric cancer cells [16].

The study by Pan et al., who worked on the gastric cancer cell line SGC7901, showed that SEMA5A was significantly overexpressed in these cells and effectively invades them [17]. Then, siRNAs decrease SEMA5A expression, which significantly reduces angiogenesis and metastasis in these cells; on the contrary, it increases the apoptosis induction. Another intriguing study again by Pan et al. showed that SEMA5A could affect MMP-9 by acting on the MEK/ERK pathway and could play a key role in gastric cell invasion [18]. Another study revealed the SEMA5A important role in metastasis and invasion of gastric cancer cells by uPA regulatory activity and the PI3K/Akt pathway [19]. These studies showed that the semaphorin family of proteins play a key role in the nervous system and can also be effective in the cancer development and progression.

To select a suitable biomarker with a bioinformatics approach, it is better to evaluate the high-expressed genes more carefully and observe which protein products of these genes release into the extracellular matrix [20]. Then, in the next steps, the path and the mechanism of these genes or their protein products can be confirmed, and the appropriate strategies can be achieved for timely detection. This study selected the BGN, LOX, MMP-9, SERPINE1, and TGFB1 proteins with more and more accurate relationships in different signaling pathways based on this method. The drawn protein network shows the critical connection between these proteins and other proteins.

Lysyl oxidase (LOX) was one of the selected proteins in this study. It plays a vital role in organizing the ECM by regulating posttranslational processes in fibrous proteins, such as elastin and collagen [21]. Several studies have examined LOX in gastric cancer. Zhao et al. identified a significant association between LOX and MMP-2 and MMP-9 in the gastric cancer cell line and tumor tissue. Decreased expression of MMP-2 and MMP-9 was also observed in the presence of inhibited LOX [22]. The study by Han et al. also showed that increased LOX expression was directly related to HIF-1; increased expression of this gene indicated the hypoxia role in increasing the activity of tumor cells in gastric cancer [23].

Kasashima et al. expressed the inhibition of LOX by siRNAs could reduce E-cadherin expression and increase vimentin expression, thereby activating the EMT pathway [24]. The EMT pathway is one of the most key and effective pathways to enhance the growth, proliferation, and invasion of tumor cells [25]. Zhang et al.'s study also showed that even after surgery in patients with gastric cancer and tumor tissue isolation, LOX expression was high, which led to the recurrence of the disease [26]. Based on the bioinformatics analysis in this study, it was also shown that LOX expression was significantly higher in patients with gastric cancer. Also, according to the survival chart, individuals' survival decreases significantly over time, which might indicate the key role of this gene in the invasion of gastric tumor cells.

Another studied gene was BGN. It is a member of the proteoglycan group and is leucine rich, which plays a role in regulating fibrous proteins such as elastin and collagen fibers [27]. A study by Hu et al. showed that high BGN expression could play a role in metastasis and invasion of gastric cells into lymph nodes in both laboratory and animal phases. To induce gastric cell invasion, BGN also stimulates the FAK/paxillin signaling pathway, which significantly increases metastasis [28]. Other studies have been performed on BGN and gastric cancer, most notably in bioinformatics analysis; they have related gastric cancer patients with healthy individuals. High expression of BGN is found in gastric cancer [29, 30]. The present study examined the noninvasive to invasive stage of gastric cancer; BGN was found by examining the ECM proteins. As one can see in Figure 5, the survival graph for BGN also decreases dramatically over time.

SERPINE1 in the extracellular matrix revealed another gene association with the noninvasive to invasive stage of gastric cancer. This gene plays an essential role in the cell adhesion, migration of tumor cells to other organs, and cellular aging [31]. The study by He et al. showed that SERPINE1 inhibition using long noncoding RNAs reduces the invasion of gastric tumor cells significantly [32]. Transcriptome studies have shown that SERPNE1 can play an important role in the EMT pathway [33]. Based on the survival chart obtained from this gene, it can be concluded that mortality is directly related to other candidate genes due to increased expression of SERPNE1 and mortality in gastric cancer patients.

Matrix metalloproteinase plays a significant role in the invasion of cancer cells. To date, numerous studies have proven the effective role of this family to invade cancer cells in many malignancies [34]. However, an interesting examined relationship in this study was the role of semaphorin and LOX with MMP-9; these two different signaling pathways can play an important regulatory role for MMP-9.

The study by Wang et al. also showed that MMP-9 is effectively involved in the EMT pathway in gastric cancer. When melatonin was used, the *IL-1β/NF-κB/MMP-2/MMP-9* genes' expression and also invasion of gastric cancer cells reduced significantly [35]. Due to the crucial role of MMP-9 in ECM regeneration, it seems that several factors are associated with this gene. HOXC6, as an important transcription factor, enhances the MMP-9 activity and is involved in increasing the metastasis of gastric cancer [36]. Also, the RUNX3 signaling pathway regulating TIMP3 can be effective in MMP-9 activity in gastric cancer invasion [37].

In association with TGFB1 in gastric cancer, there are limited studies on this gene's biomarker role. Most studies are about the mutations, variants, and polymorphisms of TGFB1 with gastric cancer. For example, the C-509T [38] and 509C/T [39] mutations were significantly associated with gastric cancer invasion. Therefore, in this study, by performing bioinformatics analysis, we showed that TGFB1 could also be considered as a diagnostic biomarker for gastric cancer.

## 5. Conclusion

Finally, it can be mentioned that, with the help of continuous bioinformatics analyses, BGN, LOX, MMP-9, SERPINE1, and TGFB1 proteins enhance the progression of gastric cancer; they play a significant role in the organization and communication of cells in the extracellular matrix. Also, a closer look at the mechanism of action between semaphorin, LOX, and MMP-9 can reveal new pathways in gastric cancer invasion and its association with the EMT pathway.

## Figures and Tables

**Table 1:** Biological processes in the upregulated and downregulated genes in the noninvasive and invasive stage of gastric cancer.

Biological process terms	FDR	Top 50 genes
*Upregulated genes*		
Anatomical structure morphogenesis	1.75*E* − 29	*GAS7 ADGRA2 PITX1 MAP4K4 WDR1 FRYL ISM1 HOXA2 PMP22 CDH6 COL7A1 PLXNA1 RHOQ BHLHE41 RHOJ PALLD MAP1S PLXNA3 RAMP2 ZSWIM4 BHLHE40 RAC1 TMOD1 SULF1 THBS1 PARVG RNF165 COL6A1 COL6A2 SLIT2 VEGFC THY1 PGM5 BRSK1 LRP5 COL6A3 LMOD1 HEYL NIPBL DACT1 ILK MAP1A ISLR2 LUZP1 SEMA3E*
Regulation of the cellular component organization	5.22*E* − 23	*APP UBE2J2 ARIH2 WDR1 OGFOD1 PALM VPS18 ARHGEF10 TESK1 PLXNA1 ALMS1 PLEKHM2 CCND2 RHOQ RHOJ MACF1 MAP1S PLXNA3 ZSWIM4 ARHGAP17 RHOT2 SLIT2 ASAP1 THY1 WHAMM DDR2 NIPBL NDEL1 MAP1A ISLR2 RAB4B SEMA3E CLSTN1 RHOG CDC42EP4 H1FX ATXN2 ZSWIM8 STON1 TWF2 APOPT1 NOL12 SYT11*
Regulation of the developmental process	2.52*E* − 21	*APP ISM1 PLXNA1 RHOQ BHLHE41 CNOT1 RHOJ JUND PLXNA3 ZSWIM4 BHLHE40 SULF1 THBS1 VEGFC THY1 HEYL DACT1 NDEL1 RBPMS2 ISLR2 SEMA3E JUNB CLSTN1 RHOG APOLD1 CDC42EP4 MAFF THBS2 HES4 MAFG ENPP1 ZSWIM8 TWF2 NFKB1 HSPB6 PTBP1 DCN RNH1 FOXJ2 VASH1 NUAK1 FBLN1 YPEL3 SETD1A SYNDIG1*
Actin filament-based process	3.95*E* − 18	*GAS7 WDR1 FSCN1 SPECC1L ARHGEF10 TESK1 EPB41L5 RHOQ RHOJ LLGL1 RAC1 TMOD1 PARVG FRMD6 TPM1 ARHGAP17 IQSEC1 PGM5 WHAMM SHROOM4 LMOD1 TPM4 RHOG CDC42EP4 SMTN TPM2 AIF1 TWF2 CNN2 PPM1F MYH9 HCK CORO1A ARHGEF18 TGFB3 MYH10 MICAL1 SLIT2 DLC1 DAPK3 TYROBP MEF2A ACTN1*
Cell adhesion	3.53*E* − 17	*MYH9 MYL9 TLN1 CSRP1 ILK ITGB5 ICAM2 CDH6 PLXNA1 TGFBI MACF1 PALLD PLXNA3 EMILIN1 PARVG FER THY1 ITGB2 DDR2 FBLN2 CERCAM NLGN2 CDH5 TENM3 ZYX NUAK1 FBLN1 SPECC1L SERPINE1 VWF BVES DUSP22 THBS4 ADAM19 HAVCR2 RAC1 THBS1 ERBB2 MSN NOTCH1 SPOCK1 ITGA5 PDPN IGFBP7 DLC1 DAPK3*

*Downregulated genes*		
RNA processing	7.24*E* − 27	*NSUN2 TRIT1 THUMPD1 FTSJ1 RBM7 MBNL3 SEPHS1 PAPOLA GEMIN2 SRPK1 PES1 PHF5A DICER1 PRPF6 RIOK3 RBM3 ERI1 POP4 EXOSC3 DHX40 INTS2 FTSJ3 UTP6 LUC7L3 PUS3 NOP2 SRSF3 BYSL DROSHA NCBP2 SF3B6 SFPQ RPF1 PRPF3 PPP1R8 CPSF3 HEATR1 RBM25 EXOSC8 TRMT1L HNRNPA2B1 EXOSC9 RRP36 SNRPC AARS2 RIOK1 RNF113A TRMT5 NGDN*
Organonitrogen compound biosynthetic process	1.40*E* − 24	*MRPS17 DPM1 GCLC ELOVL5 COX15 SARS FUT8 ADSS CTPS2 GATB PIGV ZC3H15 ELOVL1 IARS2 COASY FTSJ1 ASNS RPL31 HACD3 ITM2A POMGNT1 SEPHS1 RPL6 RPLP0 EIF3L RBM23 PIGH PIGU PRPS2 PGK1 SMS RBM3 HAS3 GSPT1 ZDHHC2 GARS C1GALT1 EIF3A PNPO UGDH GAPDH TPI1 HDDC2 MTRF1L MRPL2 TARS RARS SPR CTH WARS2 RPN2 ALG5 ETF1 SERP1 MTIF3*
Mitotic cell cycle	9.63*E* − 24	*PAFAH1B1 ANLN PHF23 TUBE1 NDC80 PDS5B ZW10 PEBP1 BIRC5 KIF4A MSH2 RANBP1 PSME2 POLA1 NBN CCNJ BCCIP TUBD1 DUSP3 UNC119 NCAPG ANAPC15 CDKN1B CCNG1 NEK11 MSH6 NEK2 NSL1 NAA50 KIF18A CDK2 CKS2 MSTO1 STK33 CCNB1 USP26 DSCC1 TUBB2A NUSAP1 KIF23 KIF11 CEP55 CCNG2 CENPE CLTC ANAPC11 NUF2 CENPC DSN1 TUBA3E*
Organelle organization	1.09*E* − 22	*TUBB2A NUSAP1 KIF23 RSL24D1 KIF11 MNS1 USP8 NUP54 CCNG2 CENPE UQCRC2 AFG3L2 ESCO1 ANAPC11 PLK4 MTF2 NUF2 GOLPH3L RHOB EML4 LNPK HSPD1 DYNC1LI1 CENPC ABT1 PHIP ARHGAP18 ADD3 DSN1 TWF1 TUBA3E PSTPIP2 HAUS1 TADA1 CCNO SAR1B ANAPC1 INO80C TRIP12 BUB3 EME1 NDUFAF6 WHAMM PCGF6 UQCRB MTERF3 BUB1B TMED6*
Cellular response to stress	4.35*E* − 21	*RECQL SPAG9 SPRTN MAP4K3 BRCA1 ERCC1 MBTPS2 MAP4K5 HSF2 BAK1 MAP2K3 GABARAPL2 ERCC8 RAD51 PMS1 SLK POLB MLH1 UNG TRAF4 UBE2T XRCC5 HSP90AA1 PDS5B MSH2 MAP3K1 CDC7 PSMC6 POLA1 NBN UBR5 GSR RPA3 ERLIN1 NSMCE4A BCCIP MAP3K8 DUSP3 MAP2K6 HSPA8 UBE4A TDP2 ASCC3 HSPA9 OGG1 NEK11 AUP1 NFE2L2 MSH6 PRDX1*

## Data Availability

We selected the appropriate datasets from the GEO database. We evaluated the genes' signaling pathways, biological processes, and molecular functions more accurately.
